# The peer-review process in Europe’s leading case report journal for cardiovascular diseases: a survey for improving transparency and quality

**DOI:** 10.1093/ehjcr/ytae118

**Published:** 2024-03-04

**Authors:** Edoardo Conte, Philipp Sommer, C Fielder Camm, Monika Radike

**Affiliations:** Clinical Cardiology and Cardiovascular Imaging Unit, Galeazzi-Sant’Ambrogio Hospital IRCCS, via Cristina Belgioioso 173, 20100 Milan, Italy; Clinic of Electrophysiology, Herz-und Diabeteszentrum NRW, Ruhr-University Bochum, Georgstraße 11, 32545 Bad Oeynhausen, Germany; Keble College, University of Oxford, OX1 3PG Oxford, UK; Department of Radiology, Liverpool Heart and Chest Hospital, Thomas Drive, Liverpool, UK; Liverpool Centre for Cardiovascular Science, Department of Radiology, Liverpool, UK

## Abstract

**Figure:**
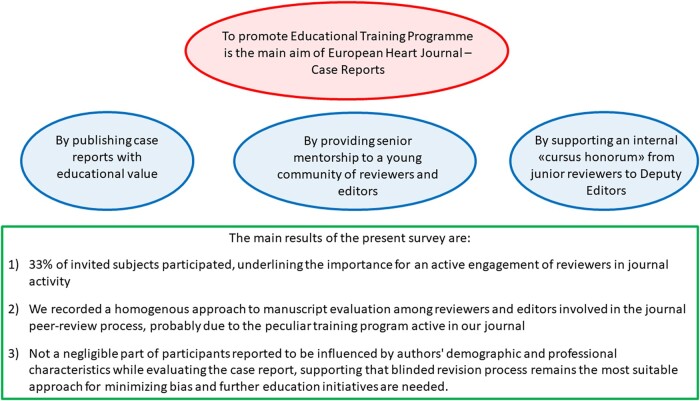
Graphical abstract summarizing main study results.

Graphical abstract summarizing main study results.

## Introduction

The number of cardiovascular journals publishing scientific articles after the peer-review process has increased dramatically in the last decades.^1^ As a consequence, an increasing number of healthcare professionals, mainly physicians at different stages of their professional careers and from different regions in the world, have been involved in the publishing process both as authors and peer reviewers and in more limited cases as editorial board members. This has several positive effects such as expanding the community of physicians interested in academia. The peer-review process is a fundamental characteristic of the modern way of reporting scientific results, not only in the medical field and this process should be as much as possible free of bias and focused on the quality of scientific results. However, the involvement in the peer-review process of non-‘professional’ researchers, with less expertise in clinical research practice and rules, may undermine the quality and accuracy of the publishing process that is crucial to keep the quality of academic publications at high standards. Moreover, different career stages, clinical interests, and countries of origin may introduce differences among editors’ and reviewers’ judgments.

The EHJ-CR mission includes creating and educating a community of young reviewers and editors (clinicians and researchers in cardiovascular medicine and related fields) to help maintain the highest quality of the academic publishing process.

After several years of journal activity, as a community, we have an opportunity to interrogate ourselves on the peer-review process we are experiencing in the EHJ-CR. Accordingly, we have surveyed the journal’s editors and reviewers with the following aims:

1. To explore the composition of active and engaged EHJ-CR editorial board members, who decide to participate to the survey, in terms of demographics, career stage, and main clinical interest;
2. To explore, in a systematic approach, what is considered important by the editor and reviewer in the terms of both content and formal presentation of the manuscript;
3. To explore what is considered as fundamental in authors’ response by the editors and reviewers during each different stage of the review process; and
4. To explore on potential pitfalls of peer-review process that should be eventually monitored and addressed in order to keep high the peer-review process quality.

## Methods

An international survey targeting Deputy Editors, Associate Editors, and Reviewers of EHJ-CR that was developed using an external platform (Google Forms, Google LLC, CA, USA) was adopted, and invited subjects received a link to fill out an electronic form. The survey consisted of 48 questions divided into four sections as follows: the first, is dedicated to demographic and professional data; the second, is dedicated to explore editors’ and reviewers’ first approach to the manuscript; the third, is dedicated to explore editors’ and reviewers’ approach to the resubmitted manuscript after revision; and the fourth is dedicated to specifically explore and identify the prevalence of potential pitfalls that may affect the peer-review process such as whether demographical and professional characteristics of the authors may impact editor and reviewer approach to the manuscript. These sections are consistent with the pre-specified aims of the survey. The questionnaire is presented as Supplementary material to the present manuscript. The survey was disseminated to EHJ-CR collaborators via group emails. Regarding the selection of subjects to be involved in the present survey, it should be underlined that, due to its educational mission, EHJ-CR benefit from a list of engaged reviewers, beyond Deputy and Associate Editor, who agreed to regularly collaborate with the Journal and were thus invited to complete the survey. Similarly, Associate Editors agreed to serve as reviewers for the Journal on a regular basis.

## Results

Out of 375 invitees, a total of 126 (33.3%) participants adhered to the present survey and all of them filled out the entire form without missing data. For what concern ethnicity, most of the participants self-defined as white (96 subjects, 76.2%) or Asian (19 subjects, 15.1%) with other ethnicities under-represented. Among them, 8 (6.3%) were Deputy Editors, 53 (42.1%) were Associate Editors, and 55 (43.7%) had a Reviewer role in the Journal.

### Demographics and professional data

**Figure 1:**
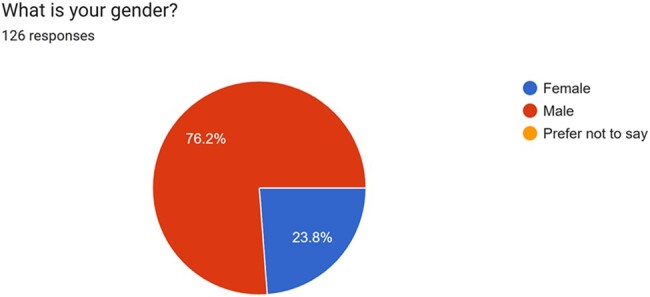
Pie chart representing gender distribution across survey respondents.

The 126 Journal collaborators involved in the present survey had a mean age of 38 ± 7 years old and were predominantly male (96 male participants, 76.2%), (*Figure 1*), with 76 participants from the EU and 50 from non-EU countries. For what concern professional positions, the majority of participants was early-career physicians (42.1%) with a lower prevalence of Full Professors (9.5%). The great majority of those involved in the survey was cardiologist specialists (90.5%) with all major subspecialties areas well represented (20.6% heart failure specialists, 15.9% cardiac imagers, 19.8% dedicated to electrophysiology, and 17.5% interventional cardiologist). Other specialties included were cardiac surgeons (4.8%), internal medicine specialists (1.6%), and radiologist (0.8%).

### The first approach to the manuscript

**Figure 2:**
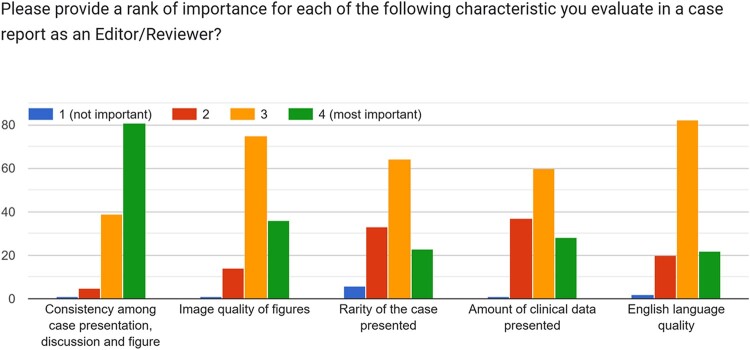
Bar chart representing case report characteristics importance according to editor/reviewers.

**Figure 3:**
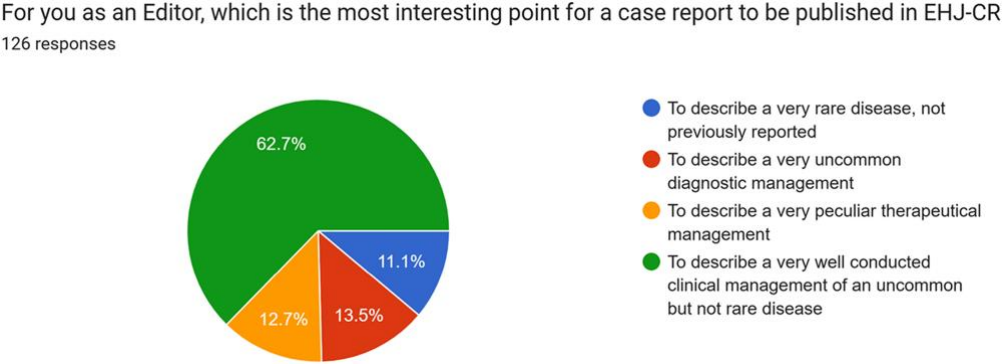
Pie chart representing most interesting points of a case report according to reviewers/editors.

**Figure 4:**
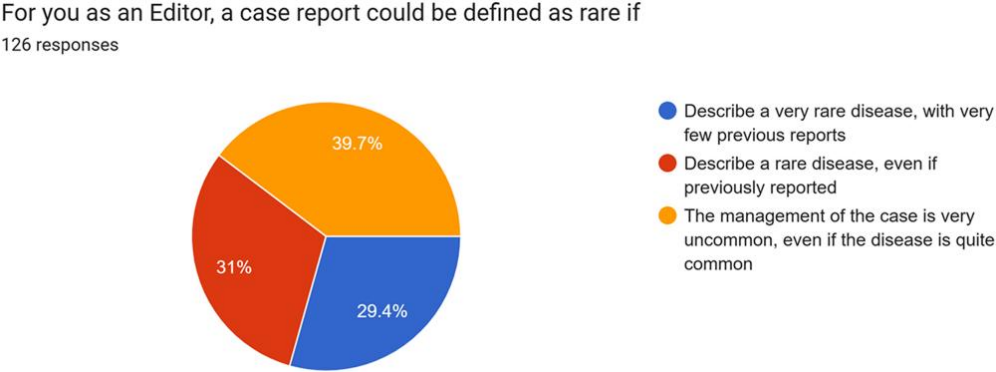
Pie chart representing distribution of different definition of ‘rarity’ of a case report according to editors.

The first manuscript evaluation is crucial for determining the potential interest for publication in the journal. Data from the present survey suggested that one of the most important features a case report should have for being considered is the consistency among case presentation, discussion, and figures (*Figure 2*); similarly, the clarity of the clinical message in a case report was considered as most important by 92.1% of participants. These were indicated as the two main characteristics a case report should have by 69.8% and 76.2% of participants, respectively. For the majority of the Editors (62.7%) of the Journal, ‘to describe a very well conducted clinical management of an uncommon but not rare disease’ emerged as the most important characteristic for a case report to be considered for publication (*Figure 3*). Of interest, the majority (69.8%) of subjects involved in the survey did not consider as of ‘most importance’ that procedures described in the case report strictly adhere to international guidelines. Similarly, only 18.3% of subjects indicated the rarity of the disease presented as one of the most important features the case should have for being considered for publication and only 14.3% considered the presence of clinical follow-up as ‘most important’ for an interesting case report. The present survey underlined a quite disagreement among on what defines a case report as rare, as detailed in *Figure 4*, while 51.6% of participants agreed that the case report title should clearly inform readers regarding the final diagnosis.

For what concern figures quality, the presence of ‘A very clear message to be conveyed to the readers’ resulted as one of the most important characteristics for the majority of participants (90 out of 126) while both very high quality of some technical characteristic, such as spatial resolution, and the accuracy of figure legend and caption were not considered as ‘most important’ at this stage of peer-review process by the majority of participants (26 and 30 out of 126, respectively).

### The review process

The reviewer’s evaluation of the manuscript and the authors’ response to the issues raised represent the core elements of the peer-review process. The first step we explored was the editor selection and invitation of the reviewer, and most of the participants declared that the automatically provided classification matches is the most common tool used for reviewer identification. Not rarely it is reported that reviewers are selected according to personal relationships and, of note, the international reputation of the reviewer is considered as ‘important’ or ‘most important’ by 57.1% of participants; some importance is attributed to reviewer quality, according to the provided rating of previous review performed, and to accountability in terms of the number of un-assigned tasks after acceptance, with those having the lower number of the un-assigned task being considered as the most valuable reviewer. On the contrary, the great majority of subjects interviewed (91.3%) did not consider the reviewer’s country of origin among the parameters evaluated for its selections. Reviewers’ selection could be influenced by automatic matching according to previous publications, which is not available for our journal. Almost all of the reviewers interviewed reported being able to complete the case review in <5 h and more specifically 42.1% reported a mean time between 1 and 5 h while 50% between 30 min and 1 h.

For what concern the authors’ rebuttal letter, its polite tone and quality were considered as ‘important’ or ‘most important’ by 92.1% of participants; similarly, 96% of subjects reported that for a positive evaluation, authors should completely and positively address all issues previously raised by Editors or Reviewers. Most of the participants interviewed consider rejecting the manuscript after the first round of revision if the clinical data requested are still missing (54%) or the learning point is still unclear (61.9%). On the contrary, the absence of improvement in figures’ image quality is considered a major cause of rejection by <10% of reviewers. Of interest, 42.1% of reviewers reported recommending the rejection of a case report after the first round of revision in <25% of cases, and 88.1% suggested rejection after the second round in <25% of cases.

### Potential pitfalls in the peer-review process

**Figure 5:**
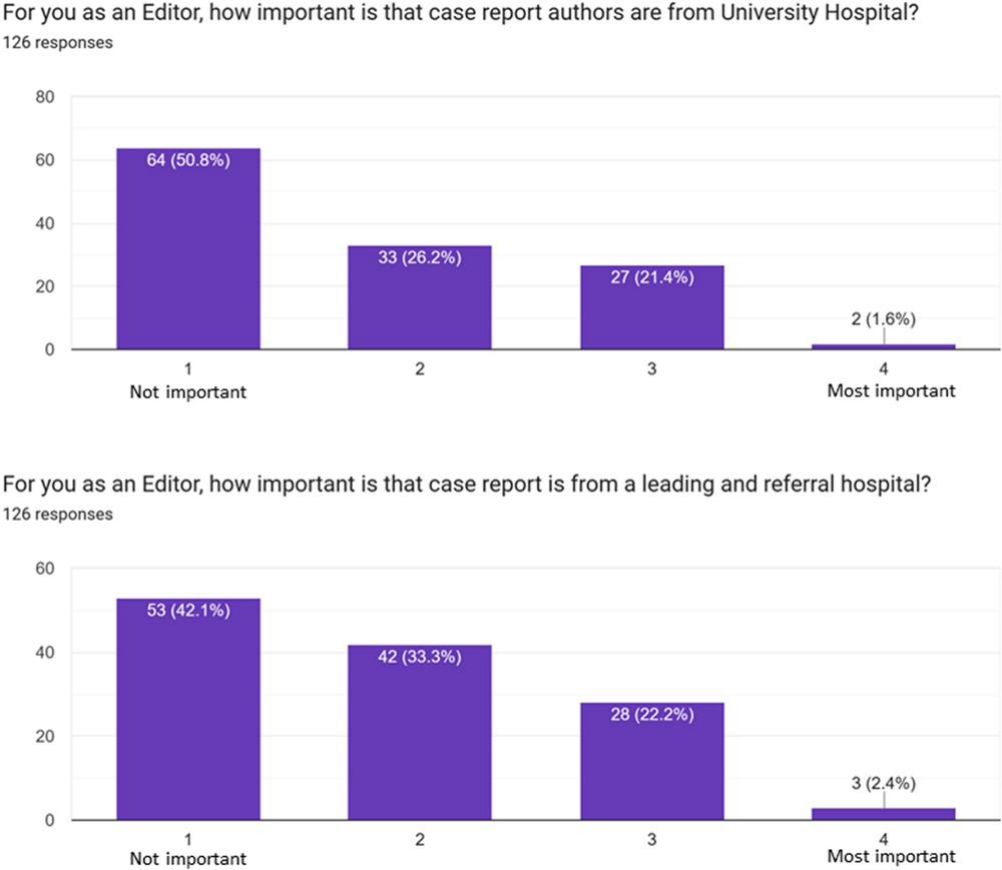
Bar chart representing potential bias in case report evaluation according to authors hospital of provenience.

**Figure 6:**
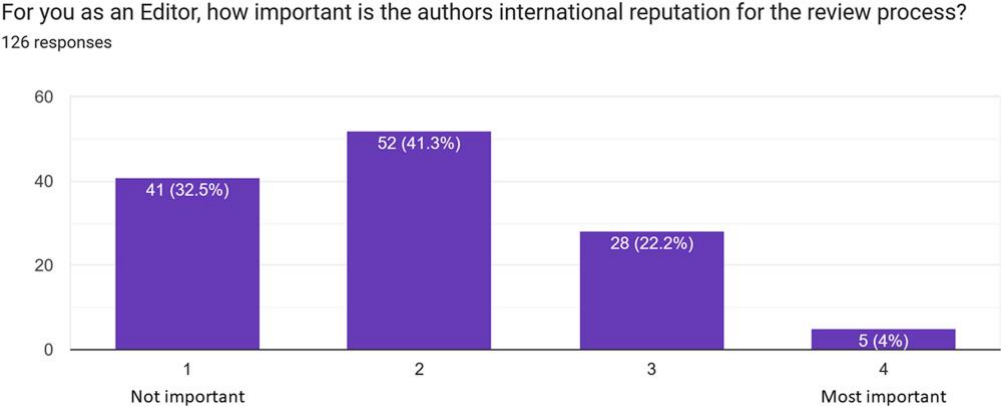
Bar chart representing potential bias in case report evaluation according authors’ international reputation.

From the present survey emerged that most reviewers/editors do not consider authors’ country of origin while evaluating a manuscript, with only 8.7% of participants reporting to consider this information. Of interest, 21.4% of subjects reported as ‘important’ that a case report comes from a university hospital, and 22.4% were favourably impressed when the authors of the case practice in a referral and well-known hospital (*Figure 5*). However, 50.8% of subjects reported as of no importance the type of hospital where the case report comes from. Similarly, author’s international reputation (*Figure 6*) is considered as ‘important’ or ‘most important’ in 22.2% and 4% of cases, respectively, and authors main specialty is evaluated and considered as ‘important’ or ‘most important’ in 33.3% and 8.7% of cases, respectively; the majority of reviewers/editors considered as ‘important’ or ‘most important’ (42.1% and 34.1%, respectively) that at least one author of the case is a physician. On the contrary, only 12.7% of participants reported to consider as ‘important’ or ‘most important’ that the first author of the case is a senior physician. Finally, 56.3% and 18.3% considered as ‘important’ or ‘most important’ to agree with the way the case was reported to be managed by authors.

## Discussion

At the best of our knowledge, the present is the first report of an internal survey among editors and reviewers of a peer-review indexed journal. A previous experience of journal internal evaluation is from the *Journal of the Egyptian National Cancer Institute* (JENCI) that performed a formal re-evaluation of articles published but no active engagement from editorial board members or reviewers was requested.^2^ Previous reports underlined potential pitfalls of the peer-review process^3^ and reported the presence of gender and geographical inequalities affecting the entire process.^4^ The present survey may represent an interesting example of journal internal self-inquiry aimed at promoting transparency and high ethical standard in the peer-review process.

The initial crucial finding is that only 33% of the invited participants actively engaged in the survey. This highlights that, even in a journal emphasizing ‘educational values’ and boasting a roster of reviewers affiliated, active involvement in journal activities is relatively low. While a more dedicated subset of editors and reviewers may be more engaged, it should be underlined that many journals require a substantial number of editors and reviewers to ensure timely evaluations of submitted papers, potentially reducing the quality of the review process.

The participants of the survey were mostly cardiologists in their early careers with a very good balance between EU vs. non-EU-based respondents (even if ‘white’ was by far the most represented self-defined ethnicity) and with all cardiology subspecialties adequately represented. Of note, most of those involved in the survey were male (76.2%), highlighting the lack of gender diversity representation. This pattern has been previously reported.^4^ Moreover, in a recent cross-sectional study, a significant gender gap in the leading posts of top-ranked medical journals was found, which underlines the importance of structured and sustainable diversity and equality programmes in academic medicine and publishing.^5^ Similarly, the low participation in journal activities of specialties other than cardiologists may have an impact on article selection in the era of multidisciplinary medicine.

The results of the present survey fulfil the educational aim of our journal, providing some insights on how editors and reviewers approach case reports submitted to EHJ-CR. Of interest, the extreme rarity of the case presented is not considered on average as one of the most important characteristics a case should have for being considered in the journal, but the consistency between the main text and figures, together with the clarity of the clinical message to be conveyed to readers are two of the most important points that positively impress reviewers and editors of EHJ-CR. This aligns with the educational scope of the journal, which publishes not only novel but also educational valuable case reports as detailed in the journal webpage. Similarly, technical image quality parameters (like spatial resolution) are not considered fundamental, while the quality and clarity of the message conveyed by the figure is one of the most important characteristics a figure for being considered as a ‘good one’.

For what concern the review process, one of the most interesting and important results of our survey is that the majority of participants declared to need from 1–5 h of work to complete a review of a case report. Even taking into consideration the relatively young age of colleagues involved in our survey, this underlines how accepting to act as a reviewer for a journal could be a time-consuming task. In the scientific community, there have been discussions about the peer-review process and how, and if it should be compensated.^6–8^ In this regard, some of the physicians involved in the survey support the introduction of some kind of compensation for the reviewer role such as being invited to write an editorial article for the journal without article publishing charges, as a direct consequence of accepting the reviewer role. In this regard, other journals offer compensation items such as vouchers of free full-text access to the publisher system and monetary vouchers for books. These incentives may have the potential to enhance the quality of the reviewer process, thereby encouraging accomplished researchers to willingly take on the role of reviewers.

Overall, we recorded a homogenous approach to manuscript evaluation among reviewers and editors involved in the journal peer-review process. This was promoted by several educational initiatives that the editorial board encouraged in the past and that were disseminated through all professionals involved in the peer-review process; these data may support the value of EHJ-CR model that advocates for a fixed journal team of not only editors but also reviewers to promote a shared and reproducible approach to the manuscript evaluation process.

Of interest, not a negligible part of participants reported to be influenced by authors’ demographic and professional characteristics while evaluating the case report. This is to be seen in the context of blinded peer-review in the Journal in the case of reviewers, whereas the editors are not blinded to the author information. Even if the majority of our editors and reviewers reported not being influenced by these factors, this represents a controversial result as the reviewer and the editor should evaluate the manuscript *per se*, without being influenced by authors’ background. Although the surveyed cohort primarily comprises young and early-career professionals, the data suggest that a blinded revision process remains the most suitable approach for minimizing bias. Moreover, these findings may prompt leaders of editorial boards to address this aspect more effectively in educational meetings focused on raising awareness and managing unconscious bias.^9^ This reinforces the value of our survey initiative in enhancing the overall quality of the journal. Further research is needed to answer the question of whether there is an actual (perceived or implicit) bias in peer review and editorial decisions.

### Limitations

We are aware that the present survey could be limited in its applicability to journals with different scopes such as research trials, and case reports being the main topic of EHJ-CR. Moreover, it should be underlined that participants to the present survey were mostly cardiologists, with under-representation of other specialties. However, we see it as an exploratory work on editorial board members self-interrogation on their review process: we believe that this approach could be easily translated to different settings.

## Conclusions

The present is the first report of an internal survey among editors and reviewers of an indexed and peer-review journal regarding their methods of approaching a manuscript. We recorded a homogenous approach to manuscript evaluation among reviewers and editors involved in the journal peer-review process, but not a negligible part of participants reported to be influenced by authors’ demographic and professional characteristics while evaluating the case report. This transparent and self-evaluating approach could be extended to other traditional journals to improve the peer-review process quality that could be considered the pillar of evidence-based medicine.

## Supplementary Material

ytae118_Supplementary_Data

## Data Availability

The data underlying this article will be shared on reasonable request to the corresponding author.

## Supplementary material

**Funding:** None declared.

## Editor statement

We confirm that the paper was handled by an independent Editor as the EiC is among the authors.
