# Supplementary material for: The peer-review process in Europe’s leading case report journal for cardiovascular diseases: a survey for improving transparency and quality
Source: Eur Heart J Case Rep. 2024 Mar 4;8(3):ytae118. doi: 10.1093/ehjcr/ytae118 (PMC10957160; doi:10.1093/ehjcr/ytae118)
Supplement: ytae118_Supplementary_Data [file ytae118_supplementary_data.pdf]

EHJ CR Editor survey

Dear Editors and Reviewer,
You are being invited to participate in a survey for the EHJ Case Report
The number of cardiovascular journals publishing scientific articles after peer review process have increased dramatically in the last decades. Of consequence, an increasing amount of healthcare professionals, mainly physicians at different stage of their professional careers, have been involved in the peer review process both as authors and reviewers and in more limited cases as editorial board members.
This process have several positive effects such as to expand the community of physician interested in clinical research. However, the involvement in the peer review process of non "professional" researchers, with less expertise in clinical research practice and rules, may undermine the quality and accuracy of the peer review process that is crucial to keep the quality of clinical research at higher standards.
The EHJ-CR mission includes to create and educate a community of young reviewers and editors (and researchers in cardiovascular medicine) that could be ready in the future to keep the quality of the peer review process at the highest level.
After several years of journal activity, as a community, we consider the opportunity to interrogate ourselves on the peer-review process we are experiencing in the EHJ-CR.
Accordingly, we promote the present survey with the following aims:
- To explore the composition of EHJ-CR editorial board in term of age, sex, region of activity, career stage and main clinical interest.
- To explore what is considered important by the editor and reviewer for the final decision on article acceptance in the terms of both content and formal presentation of the manuscript
- To analyze whether the community of editors share a common feeling on what is needed for a case report to be accepted in the journal and eventually to identify non-classical and potentially unfair behaviors affecting the decision processes
- To inform reader and potential authors on what is considered important by the editorial board in order to improve the quality of case report submitted at different stage of the review process.
- To discuss on potential pitfalls of peer-review process that should be eventually monitored and corrected in order to keep high the peer-review process quality.
We are aware that being case reports the main topic of EHJ-CR the present survey could be limited in its applicability to journals publishing research trial; however, this could be one of the first example of editorial board members self-interrogation on their review process that could be replicated in different settings.

We believe there are no known risks associated with this study; however, as with any online related activity the risk of a breach is always possible. We will minimise any risks by not asking any identifying information, key coding the data for processing and destroying the raw data after acquisition is completed.
If you have any queries, you can email at edoardo.conte86@gmail.com.
Thank you very much.
Dr Edoardo Conte
Dr Monika Radike (neé Arzanauskaite)

* Indicates required question

FIRST AIM: to explore the demographics of survey participants

1. Please specify your main role within the Journal: *

Mark only one oval.

- Associate Editor
- Deputy Editor
- Reviewer
- Other: ____________________

2. What is your age?

____

3. What is your gender? *

Mark only one oval.

- Female
- Male
- Prefer not to say

4. Please specify your ethnicity *

Mark only one oval.

- Asian
- Black, Black British, Caribbean or African
- Mixed or multiple ethnic groups
- White
- Other: ____________________

5. Where do you live and work? (please specify both city and country) *

____

6. Which of the following best fits your work position? *

Mark only one oval.

- ⬭ MD/PhD Student
- ⬭ Non-physician post-doc
- ⬭ Early career clinician
- ⬭ Senior clinician
- ⬭ Researcher
- ⬭ Assistant Professor
- ⬭ Full Professor

7. If you are a physician, which is your main specialty *

Mark only one oval.

- ⬭ Cardiology
- ⬭ Cardiac surgery
- ⬭ Radiology
- ⬭ Anesthesiology
- ⬭ Internal Medicine
- ⬭ Vascular surgery
- ⬭ Other: ______________________

8. If you are a cardiologist, which is your main subspecialty *

Mark only one oval.

- Cardiac Imaging
- Cardiovascular Prevention
- Acute cardiac care
- Heart Failure
- Electrophysiology
- Interventional Cardiology
- Adult Congenital Heart Disease
- Pediatric
- Not cardialogist
- Other: ______________________

SECOND AIM: to explore the first approach to the manuscript

9. Please provide a rank of importance for each of the following characteristic you evaluate in a case report as an Editor/Reviewer? *

Mark only one oval per row.

	1 (not important)	2	3	4 (most important)
**Consistency among case presentation, discussion and figure**	◯	◯	◯	◯
**Image quality of figures**	◯	◯	◯	◯
**Rarity of the case presented**	◯	◯	◯	◯
**Amount of clinical data presented**	◯	◯	◯	◯
**English language quality**	◯	◯	◯	◯

10. For you as an Editor/Reviewer, how important is the accurateness of the title in a case report *

Mark only one oval.

	1	2	3	4	
Not	◯	◯	◯	◯	Most important

11. For you as an Editor/Reviewer, how important is the clarity of the clinical message in a case report *

Mark only one oval.

	1	2	3	4	
Not	◯	◯	◯	◯	Most important

12. For you as an Editor/Reviewer, how important is good English quality in a case report *

Mark only one oval.

	1	2	3	4	
Not	◯	◯	◯	◯	Most important

13. For you as an Editor/Reviewer, how important is in a case report consistency among case presentation, discussion and figure *

Mark only one oval.

	1	2	3	4	
Not	◯	◯	◯	◯	Most important

14. For you as an Editor/Reviewer, how important is the presence of a clear diagnosis in a case report: *

Mark only one oval.

	1	2	3	4	
Not	◯	◯	◯	◯	Most important

15. For you as an Editor/Reviewer, how important is the presence of a clear learning point in a case report: *

Mark only one oval.

	1	2	3	4	
Not	◯	◯	◯	◯	Most important

16. For you as an Editor/Reviewer, how important is good image quality in a case report *

Mark only one oval.

	1	2	3	4	
Not	◯	◯	◯	◯	Most important

17. For you as an Editor/Reviewer, how important is the presence of a complete figure legend in a case report *

Mark only one oval.

	1	2	3	4	
Not	◯	◯	◯	◯	Most important

18. For you as an Editor/Reviewer, how important is the strict adherence to international guidelines in the clinical course of a case report *

Mark only one oval.

	1	2	3	4	
Not	◯	◯	◯	◯	Most important

19. For you as an Editor/Reviewer, how important is the presence of clinical follow-up in a case report *

Mark only one oval.

	1	2	3	4	
Not	◯	◯	◯	◯	Most important

20. For you as an Editor/Reviewer, please indicate the two most important points a case for reaching publication priority in EHJ-CR: *

Tick all that apply.

- ☐ Consistency among case presentation, discussion and figure
- ☐ Image quality of figures
- ☐ Rarity of the disease presented
- ☐ Rarity of the case management
- ☐ Amount of clinical data presented
- ☐ English language quality
- ☐ Accurateness of the title
- ☐ The presence of a clear learning point
- ☐ The presence of a clear diagnosis
- ☐ Completeness of Figure Legends, Tables etc..
- ☐ Other: ____________________

21. Please rank the importance of these figure characteristics you evaluate in a figure most important point for a figure to be considered of good quality is: *

Mark only one oval per row.

	1 (not important)	2	3	4 (most important)
**Excellent image quality in terms of spatial resolution**	⬭	⬭	⬭	⬭
**A very clear message should be conveyed to the readers**	⬭	⬭	⬭	⬭
**The figure should be very well integrated within the message of the manuscript**	⬭	⬭	⬭	⬭
**Figure should be complete in terms of figure legend and caption**	⬭	⬭	⬭	⬭

22. For you as an Editor, a case report could be defined as rare if *

Mark only one oval.

- Describe a very rare disease, with very few previous reports
- Describe a rare disease, even if previously reported
- The management of the case is very uncommon, even if the disease is quite common

23. When an atypical presentation of a common disease is reported: *

Mark only one oval.

- Gold standard exam for definite diagnosis must be reported for case publication
- Gold standard exam for definite diagnosis could be avoided even if feasible, but not performed
- Gold standard exam for definite diagnosis could be avoided only when not feasible

24. A case report title should ideally *

Mark only one oval.

- Clearly inform readers regarding the final diagnosis
- Inform readers regarding the clinical presentation but not the final diagnosis
- Avoid informing readers regarding the content of the case
- Attract readers without informing regarding the content of the case (Cryptic title)

25. For you as an Editor, which is the most interesting point for a case report to be published in EHJ-CR *

Mark only one oval.

- To describe a very rare disease, not previously reported
- To describe a very uncommon diagnostic management
- To describe a very peculiar therapeutical management
- To describe a very well conducted clinical management of an uncommon but not rare disease

THIRD AIM: to explore the approach to a reviewed and resubmitted manuscript

26. How important are the tone and quality of response in the evaluation of a rebuttal letter during revision: *

Mark only one oval.

	1	2	3	4	
Not	◯	◯	◯	◯	Most important

27. How important is that authors completely and positively address all issues raised by Editor/Reviewers in the evaluation of a rebuttal letter during revision *

Mark only one oval.

	1	2	3	4	
Not	◯	◯	◯	◯	Most important

28. A good authors response after the first round of revision should *

Mark only one oval.

- Include changes both in the manuscript and in the rebuttal letter
- Include changes in the manuscript as tracked changes, with authors comments in the rebuttal letter
- Include changes only in the manuscript while the rebuttal letter should be very short.

29. As an Editor or Reviewer, please indicate two main reasons for rejecting after the first round of revision *

Tick all that apply.

- [ ] Image quality that was not improved by authors
- [ ] Clinical data still missing
- [ ] Grammar and typos are still present
- [ ] Bad quality and tone of the rebuttal letter, with unclear answers
- [ ] The learning point is still unclear
- [ ] The main diagnosis is still unclear
- [ ] Figures legends/Tables are still not complete

30. How often do you recommend rejection a case report after the first round of revision: *

Mark only one oval.

- Less than 25% of cases
- From 25% to 50% of cases
- From 50% to 75% of cases
- More than 75% of cases

31. How often do you recommend rejection a case report after the second or later round of revision: *

Mark only one oval.

- Less than 25% of cases
- From 25% to 50% of cases
- From 50% to 75% of cases
- More than 75% of cases

32. As an Editor, how often you send the case to a new reviewer after first round of revision if you did not appreciate the quality the first revision? *

Mark only one oval.

- Less than 25% of cases
- From 25% to 50% of cases
- From 50% to 75% of cases
- More than 75% of cases

33. As an Editor, how important is the country of origin in reviewer selection *

Mark only one oval.

	1	2	3	4	
Not					Most important

34. As an Editor, how important is for you that the reputation of the reviewer during selection: *

Mark only one oval.

	1	2	3	4	
Not					Most important

35. As an Editor, please describe what would you like to know about potential reviewer that is not available at the moment (in less than 100 words) *

36. As an Editor, please describe the process you follow for reviewer selection (in less than 100 words) *

37. Please describe in less than 100 words how an excellent revision letter should be *

FOURTH AIM: To explore on potential pitfalls of the peer-review process

38. For you as an Editor, how important is that case report authors are from University Hospital? *

Mark only one oval.

	1	2	3	4	
Not	◯	◯	◯	◯	Most important

39. For you as an Editor, how important is that case report is from a leading and referral hospital? *

Mark only one oval.

	1	2	3	4	
Not	◯	◯	◯	◯	Most important

40. For you as an Editor, how important is that the first author of the case report is a senior physician? *

Mark only one oval.

	1	2	3	4	
Not	◯	◯	◯	◯	Most important

41. For you as an Editor, is the country from which the case report comes important for the selection process? *

Mark only one oval.

	1	2	3	4	
Not	◯	◯	◯	◯	Most important

42. For you as an Editor, how important is the authors international reputation for the review process? *

Mark only one oval.

	1	2	3	4	
Not	◯	◯	◯	◯	Most important

43. For you as an Editor, how important is the authors main specialty? *

Mark only one oval.

	1	2	3	4	
Not	◯	◯	◯	◯	Most important

44. For you as an Editor, how important is that at least one authors is a physician? *

Mark only one oval.

	1	2	3	4	
Not	◯	◯	◯	◯	Most important

45. For you as an Editor/Reviewer, how important is for you to agree with the clinical management for supporting a case report publication? *

Mark only one oval.

	1	2	3	4	
Not	◯	◯	◯	◯	Most important

46. As a reviewer, on average, how long does it takes you to complete the first review of a case report : *

Mark only one oval.

- ◯ less than 30 minutes
- ◯ between 30 minutes and 1 hours
- ◯ between 1 and 5 hours
- ◯ between 5 and 10 hours
- ◯ more than 10 hours

47. As an Editor, on average, how long does it take you to make a final decision after first round of revision: *

Mark only one oval.

	1	2	3	4	
Not	◯	◯	◯	◯	Most important

48. Any other comments *

This content is neither created nor endorsed by Google.

Google Forms
